# Navigating the Hormonal Labyrinth: Understanding the Impact of Menstrual Cycle Dynamics on ADHD Symptoms

**DOI:** 10.1192/j.eurpsy.2025.2398

**Published:** 2025-08-26

**Authors:** M. Cameira, P. Abreu, M. Pereira, M. Soares, I. Vidó, I. Lopes, F. Ramalheira

**Affiliations:** 1 Hospital Júlio de Matos - ULS São José, Lisboa; 2 Hospital do Barreiro - ULS Arco Ribeirinho, Barreiro; 3 Hospital Júlio de Matos - ULS São José, Lisbon, Portugal

## Abstract

**Introduction:**

Attention-deficit/hyperactivity disorder (ADHD) is a prevalent neurodevelopmental disorder characterized by significant sex differences in symptomatology, prevalence rates, and associated developmental challenges. Research indicates that these disparities are not merely superficial but are rooted in complex biological, psychological, and social factors. Despite the growing recognition of these differences, the underlying etiological mechanisms remain inadequately explored.

**Objectives:**

This study aims to propose a framework addressing hormonal fluctuations in females with ADHD, emphasizing the cyclical nature of ovarian hormones and their impact on executive functioning and behavioral regulation. We hypothesize that hormonal changes exacerbate ADHD symptoms during specific menstrual cycle phases, ultimately enhancing our understanding of sex differences in ADHD and informing future research and treatment strategies.

**Methods:**

We conducted a literature review to synthesize studies on estrogen levels, executive function, and ADHD symptoms. Our focus was on the role of estradiol (E2) in cognitive functions, particularly in the prefrontal cortex, and the effects of cyclical hormonal changes on behavior and cognition in females with ADHD during adolescence and the menstrual cycle.

**Results:**

Evidence suggests that estrogen is crucial for cognitive control, with fluctuations in hormone levels impacting mental performance in women. Notably, ADHD symptoms are more likely to manifest during periods of rapid estrogen decline, particularly within the menstrual cycle. These hormonal decreases correlate with reduced executive function and self-regulation at two critical phases: increased risk-taking behaviors during the mid-cycle (periovulatory phase) and heightened avoidance and negative emotions in the perimenstrual phase. Research indicates that drops in estradiol (E2) can lead to significant increases in inattention and hyperactivity-impulsivity symptoms, especially in young adult women with high impulsivity traits. Additionally, the organizational effects of puberty may interact with hormonal changes, particularly in females with advanced limbic system development, increasing the risk of emotional dysregulation and impulsive behavior. Changes in the limbic system, essential for emotional processing and memory, further underscore the importance of considering individual sensitivity to hormonal variations.

**Conclusions:**

This framework emphasizes the importance of hormonal influences in diagnosing and treating ADHD in females. By recognizing the relationship between hormonal fluctuations and ADHD symptoms, particularly via the Multiple Hormone Sensitivity Theory, we advocate for a tailored treatment approach. Future research should focus on longitudinal studies to deepen understanding and develop targeted interventions, thereby improving ADHD management and quality of life for females.

**Disclosure of Interest:**

None Declared

